# Na-TiNT Nanocrystals: Synthesis, Characterization, and Antibacterial Properties

**DOI:** 10.1155/2022/2302943

**Published:** 2022-01-30

**Authors:** Enzo V. H. Agressott, Mauro M. Oliveira, Thiago S. Freitas, Raimundo L. S. Pereira, Ana R. P. Silva, Rafael P. Cruz, Antonia T. L. Santos, Alexandre M. R. Teixeira, Tainara G. Oliveira, João H. da Silva, Alexandre R. Paschoal, Bartolomeu C. Viana, Paulo de Tarso C. Freire, Abolghasem Siyadatpanah, Polrat Wilairatana, Henrique D. M. Coutinho

**Affiliations:** ^1^Department of Physics, Federal University of Ceará (UFC), Pici Campus, Fortaleza-CE 60440-970, Brazil; ^2^Departament of Biological Chemistry, Laboratory of Simulations and Molecular Spectroscopy - LASEMOL, Regional University of Cariri, CEP 63105-000, Crato, CE, Brazil; ^3^Departament of Biological Chemistry, Laboratory of Microbiology and Molecular Biology - LMBM, Regional University of Cariri, CEP 63105-000, Crato, CE, Brazil; ^4^Departament of Biological Chemistry, Laboratory of Applied Micology of Cariri - LMAC, Regional University of Cariri, CEP 63105-000, Crato, CE, Brazil; ^5^Departament of Physics, Federal University of Piauí, CEP 64049-550, Teresina, PI, Brazil; ^6^Center os Science and Technology -CCT, Federal Universiy of Cariri, CEP 63048-080, Juazeiro do Norte, CE, Brazil; ^7^Interdisciplinary Laboratory for Advanced Materials- LIMAV, Materials Science & Engineering Graduate Program, Federal University of Piauí, UFPI, CEP 64049-550, Teresina, PI, Brazil; ^8^Ferdows School of Paramedical and Health, Birjand University of Medical Sciences, Birjand, Iran; ^9^Department of Clinical Tropical Medicine, Faculty of Tropical Medicine, Mahidol University, Bangkok 10400, Thailand

## Abstract

Titanium nanotubes have attractive morphological and physicochemical properties for several applications, such as high surface area, mesoporous structure, good stability, ion exchange capacity, and antibacterial property. Therefore, the field of nanotube applications is increasingly expanding, such as in solar cells sensitized by dye, photocatalysis, and antibacterial activity, among others. Therefore, a study of the antibacterial properties of sodium titanate nanotubes (Na-TiNTs) was carried out together with physicochemical characterizations, such as Raman spectroscopy which shows a peak characteristic of Na-O-Ti from nanotube-agglomerated regions. The XRD diffractogram confirmed the Raman spectra and evidenced the crystalline structure associated to Na-TiNT, which showed the characteristic peaks of the sodium trititanate crystal. SEM and TEM images showed the morphology of hollow nanotubes and forming semispherical particles. EDS shows the percentage values of each of the compounds in the Na-TiNT. The bacterial activity of the Na-TiNT was analyzed in *Escherichia coli*, *Staphylococcus aureus*, and *Pseudomonas aeruginosa*. Na-TiNT modified the activity of the gentamicin and norfloxacin antibiotics against multiresistant strains. Synergistic effects against Gram-positive *S. aureus* 10 and Gram-negative *P. aeruginosa* 15 bacteria were observed when the Na-TiNT was associated with gentamicin, reducing the concentration of this antibiotic that is required to inhibit bacterial growth. Another synergic effect was observed for *S. aureus* 10 with norfloxacin.

## 1. Introduction

Antibiotic resistance is a worldwide public health problem that continues to expand as microorganisms adapt to commonly used drugs. Thus, it is of paramount importance that new types of antimicrobial agents are discovered [1]. There are several strategies to combat the antibiotic resistance. Improving the photothermal and photocatalytic properties of the metal-organic framework (MOF) when modified using polydopamine (PDA) can efficiently kill bacteria. Under the combination of more heat and free radicals, the CuS@HKUST nanoparticles showed a broad spectrum of antibacterial activities. Zinc-doped Prussian blue enhances photothermal clearance of *Staphylococcus aureus* and promotes tissue repair in infected wounds. MOF-based photosensitive hydrogel can trap bacteria through the action of electrostatic adsorption, and the subsequent hyperthermia produced by Prussian blue nanoparticles (PBNPs) under NIR light can kill bacteria. Prussian blue nanoparticles with porphyrin MOF (PB-PCN-224) when irradiated with 660 nm red light showed efficient antibacterial agent against *Staphylococcus aureus* and its biofilm. For this reason, the discovery of new synthetic compounds or isolates from natural sources, which can be used alone or in coadministration with an antibiotic, is an important objective [2]. Due to their small size and high surface, nanosized inorganic particles have gained importance as antibacterial compounds; these features allow them to interact with microbial membranes [3]. Research studies have indicated that silver and copper nanoparticles possess antibacterial effects on various microorganisms, e.g., *E. coli* [3–6], *S. aureus* [3,4,6], *B. subtilis* [3], yeast [4], and *P. aeruginosa* [6]. However, these metals, such as gold, do not appear to be potential antibiotic candidates due to their high cost. So, finding cheaper nanoparticles with significant germicidal effects would be very important for pharmacology and medical science [7]. Thus, various transition metal oxides have been tested as an inhibitor of antibacterial activities [8–11].

Titanium oxides have attracted a lot of interest due to their perspective in photocatalysts, antibacterials, and solar cells. However, their wide bandgap (*E*_g_ = 3.2 eV), the effect of which is to respond to UV light and poorly absorb visible light, makes them limited in the antibacterial field [12–15], but there are some ways in which the absorption threshold would be extended to the visible range, e.g., by nonmetallic doping [16, 17], metal cation doping [18], surface modification with noble metals [9, 11], and semiconductor [19]. Therefore, in this study, we propose and describe the synthesis, characterization, antibacterial properties, and modulation analysis of antibiotic activity against multiresistant bacterial strains of *S. aureus*, *E. coli*, and *P. aeruginosa* of titanate nanotube microcrystals doped with sodium (Na-TiNT) obtained by the hydrothermal method. Titanium oxide has already been studied and reported previously for its photocatalytic properties; such a characteristic is focused in this work for the study and antibacterial applications when titanium oxide is doped with metal salts, sodium, Na-TiNT, which is an innovative application for nanomaterials of this type.

## 2. Experimental Procedures

### 2.1. Synthesis of Na-TiNT Nanocrystals

The titanate nanotubes were synthesized by the conventional hydrothermal method based on the synthesis reported in Kasuga et al. [20], and it was performed using the procedure adapted from Wang et al. [21], Viana et al. [22], and Marques et al. [23]. In this process, 3 g of TiO_2_ (anatase) powder was dispersed in 90 mL of a 10 mol/L aqueous sodium hydroxide (NaOH) solution and stirred for 30 minutes. The white suspension was transferred to a Teflon-lined stainless steel autoclave, taken to a greenhouse oven, and hydrothermally reacted for 96 h at 140°C. Then, it was cooled down to room temperature, and the samples were washed several times using distilled water. The obtained samples were dried at room temperature in vacuum for 24 h. All chemical reagents were used as received, without further purification process, and solutions were prepared in distilled water. TiO_2_ anatase powder was obtained from Sigma-Aldrich, and sodium hydroxide (NaOH) was obtained from Dinamica (Brazil).

### 2.2. Characterizations

#### 2.2.1. Raman Spectroscopy

Na-TiNTs in a powder sample were carried out by conventional Raman scattering. The Raman equipment used was a confocal optical microscope with an objective lens of 100 × /NA = 0.90 conjugated as a Raman spectrometer (WITec alpha300). The measurements were carried out using an argon (Ar) laser as the excitation source, a green laser of *λ* = 532 nm wavelength, and Gaussian field profile with an adjustable maximum output power of 10 mW. The spectra were acquired in a time of 1 s with a laser power of 300 *μ*W; with this low power, we avoid burning the sample and obtaining an optimal signal, and for the spectroscopic image, a scanning area of 30*μ*m × 30 *μ*m was made in the powder sample.

#### 2.2.2. X-Ray Diffraction

X-ray diffraction measurements were made using a diffractometer, model D8 Advance, Bruker, with angular variation (2*θ*) between 5° and 70°, a step of 0.02°, and a current and voltage of 40 mA and 40 kV, respectively, applied to the copper ampoule (Cu K*α*, 0.15406 nm). These measures were carried out in the Department of Engineering and Material Sciences of the Federal University of Ceará.

#### 2.2.3. Scanning Electron Microscopy and Transmission Electron Microscopy

SEM and EDX measurements were performed using the field emission microscope, Quanta FEG 50, with 10 mm WD (work distance), and TEM was performed using an electronic microscope LIBRA 120, Zeiss.

#### 2.2.4. Bacterial Strains and Compounds

Multiresistant strains of Gram-negative and Gram-positive models (*Escherichia coli* 06, *Staphylococcus aureus* 10, and *Pseudomonas aeruginosa* 15) were used in this assay, grown at the Laboratory of Microbiology and Molecular Biology (LMBM) of the Universidade Regional do Cariri (URCA). Each of the strains used is resistant to at least one of the classes of antibiotics used. All compounds used, antibiotics and product, were diluted in sterile water to a concentration of 1024 *μ*g/ml. In compounds that required previous dilution in DMSO, the maximum limits for this substance were respected according to the Clinical and Laboratory Standards Institute [24].

#### 2.2.5. Antibacterial Activity Assay and Modulation of Drug Assay

In order to determine the minimum inhibitory concentration (MIC) in the direct antibacterial activity and in the modifying effect of the Na-TiNT antibiotic activity, the broth microdilution procedure in 96-well plates was adopted. In both tests, the inoculants were prepared from 24-hour cultures of bacterial strains in HIA (Heart Infusion Agar) and subsequently diluted in 0.9% saline, with turbidity verified using the McFarland scale 0.5 (1 × 108 CFU/mL). In the test of direct antibacterial activity, the methodology proposed by Javadpour and collaborators [25] was used. The microdilution plates were filled with a solution composed by 10% BHI (Brain Heart Infusion broth) plus bacterial inoculum in a proportion of 10% of the volume. The wells were then microdiluted with Na-TiNT in concentrations ranging from 512 *μ*g/mL to 8 *μ*g/mL. In the antibiotic-modifying activity test, the methodology proposed by Coutinho and collaborators [26] was used. In this test, the wells of the plates were filled with a solution equivalent to that used in the direct activity test plus Na-TiNT in subinhibitory concentration. The plates were then microdiluted with antibiotics in concentrations ranging from 512 *μ*g/mL to 0.5 *μ*g/mL. The two tests were done in triplicate, with readings performed by the colorimetric method, using 20 *μ*L of resazurin in each well, after 24 h incubation in an oven at 37°C.

#### 2.2.6. Statistical Analysis

The geometric means of the triplicates were used as central data in the analyses ± standard deviation of the mean. For the statistical analysis, a two-way ANOVA test was performed followed by a Bonferroni post hoc test, using the statistical program GraphPad Prism 5.0. Values of *p* < 0.05 were considered significant.

## 3. Results and Discussion

### 3.1. X-Ray Diffraction (XRD)

**Figure 1:**
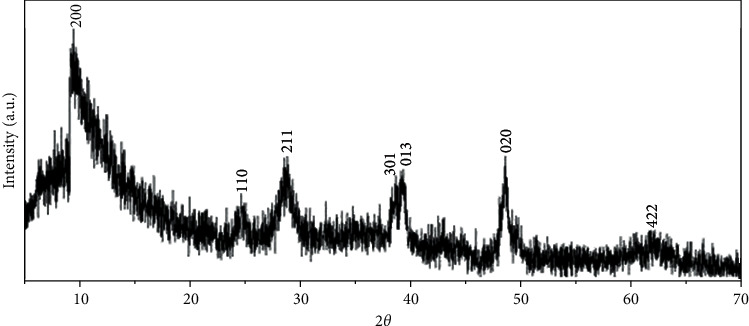
XRD of the Na-TiNT showing the characteristic shape of nanotube diffraction patterns.

The X-ray diffraction (XRD) pattern showed a typical pattern of Na-TiNT; see Figure 1. Even though the XRD of the Na-TiNT is not yet fully defined, with the XRDs performed here, it was possible to identify crystallographic planes. The crystalline planes of sodium trititanate (Na_2_Ti_3_O_7_) were also taken as a reference standard to have a clearer idea of the peaks of the nanotubes, (200), (110), (211), (301), (013), (020), and (422) (see Figure 1 and also [27–31]). All of the above suggest that the samples are similar, indicating that the crystalline structure and morphology of the titanate nanotubes have been preserved. Due to the type of the structure of titanate nanotubes, owing to their intrinsic tube nature in nanoscale, doped or modified, or not, the diffraction patterns of this type of nanomaterials tend to be noisy; nevertheless, very important information can be obtained. It should be noted and taken into account that the Na-TiNTs get a form of rolled, elongated, and hollow sheets that generate a weak crystallinity.

### 3.2. Raman Spectroscopy

**Figure 2:**
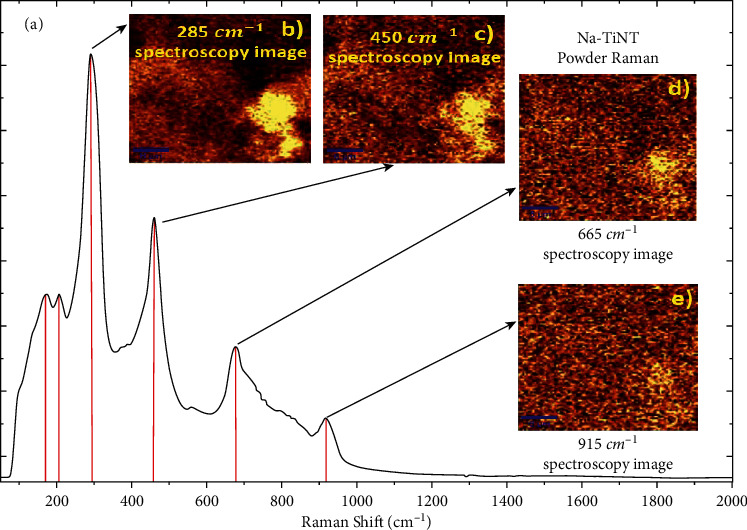
(a) Raman spectrum of the Na-TiNT, (b) spectroscopy image of the band 285 cm^−1^, (c) spectroscopy image of the band 450 cm^−1^, (d) spectroscopy image of the band 665 cm^−1^, and (e) spectroscopy image of the band 915 cm^−1^.

Figure 2 shows the vibrational spectra around 170 and 198 cm^−1^ attributed to the vibrational modes of the Na-O-Ti links; another band located around 285, 460, 665, and 705 cm^−1^ can also be observed, corresponding to the crystal photos elongation of the Ti-O-Ti junctions in the TiO_6_ octahedron; 895 and 915 cm^−1^ modes represent the stretching vibration of the Ti-O terminal bond connections not shared at the terminations of the TiO_6_ octahedron connections that involve stretching modes, which are a type of terminal bond that exits the outer walls of the nanotube and that can interact with other molecules and Ti-O-Na vibrations in the interlayer regions of nanotube walls. In Figures 2(b)–2(e), three spectroscopy images for 285, 450, 665, and 915 cm^−1^, respectively, with a full scale of 6 um can also be seen, where a spectroscopic image of the powdered agglomerates can be seen; these vibrational modes in the Na-TiNT sample indicate that the outer surface of the nanotubes is where the Ti-O bonds are found [23, 32, 33].

### 3.3. SEM, TEM, and EDS

Obtaining SEM, EDS, and TEM images of the Na-TiNT powder samples with good quality requires a very careful sample preparation. For the three characterizations, the following preparation was carried out: 0.050 g of material was placed in 50 ml of deionized water and then sonicated for 30 minutes after half of the sonicated (25 ml) was taken and another 25 ml of deionized water was added to sonicate again for another 30 minutes (the Na-TiNT liquid must be clear-transparent; if this is not achieved in the first attempt, continue repeating the process until obtaining a clear-transparent liquid). Subsequently, the final liquid is taken and passed through a syringe filter (at least 500 nm or smaller filter size if possible), and with a micropipette, a drop is deposited on a sheet of pure silicon (Si) for SEM and EDS characterization. For TEM characterization, a single drop of the Na-TiNT liquid is placed on the sample-holder grid rack and allowed to dry for 24 h and then placed on the equipment.

**Figure 3:**
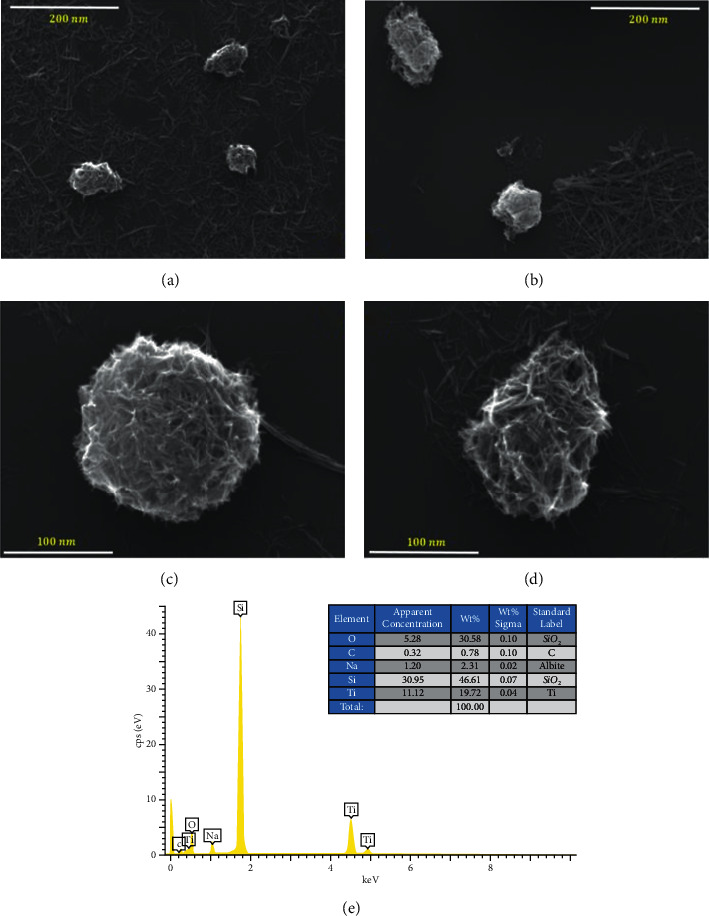
(a–d) SEM micrographs of the Na-TiNT in different regions; (e) EDS peaks for the Na-TiNT sample.

**Figure 4:**
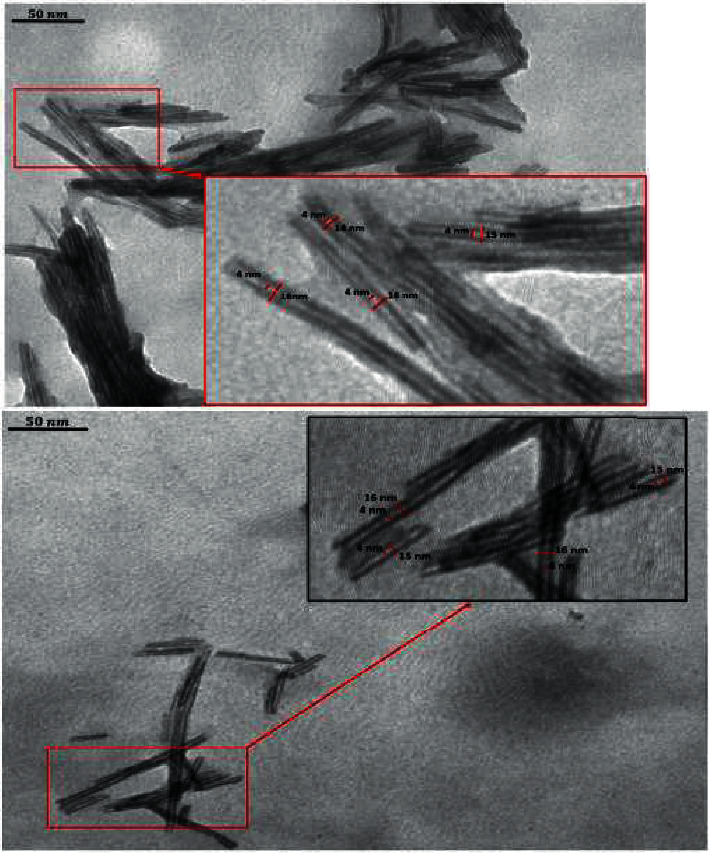
TEM micrographs for Na-TiNT samples showing the nanotubular characteristics, hollow tubular cylindrical.

From the morphological analysis obtained from the SEM and TEM images (Figures 3 and 4, respectively), it can be observed that the Na-TiNT samples have a hollow tubular cylindrical morphology and matrix nuclei (highly entangled concentrations) where nanoparticles are formed by a large number of entangled nanotubes which are agglomerated and formed, creating very porous, almost spherical nanoparticles of approximately 100 to 130 nm in size (see Figure 3); regions where there are remains of nanotubes without agglomeration can also be seen.

The great porosity of these nanoparticles means that the surface of said particles greatly increases, which makes available a large number of atoms on the surface that generate surface areas where there are active sites that make them highly efficient in active catalysts; the chemical activity is proportional to the number of active species accessible to the reagents. Therefore, it can be stated that these porous nanoparticles formed with nanotubes are highly functionalized. Several methods are used to deposit active catalysts into the pores of titanate nanotubes [34]. A method of catalyst deposition involves ion exchange of the catalyst precursor in its cationic form with protons within nanotubular titanates [35]. This allows for an atomic-scale distribution of metal cations in the titanate lattice, achieving higher metal loading compared to the adsorption of the precursor on the surface [34].

The internal and external diameters of the Na-TiNT can be estimated from Figure 4, obtaining average values between 4 and 16 nm, respectively. The internal diameter of the Na-TiNT remains constant, while the external diameter varies between 14 and 17 nm; this variation in the external diameter may be due to multiple layers.

Together with the SEM images, EDS measurements were performed; see Figure 3(e)); in this, the spectral position of the emission characteristic of X-rays was found located in *L*_*α*_ = 1,041 (Na), *L*_*α*_ = 4,452, *K*_*α*_ = 4,508 (Ti), *K*_*α*_ = 0.525 (O), *K*_*α*_ = 0.277 (C), and *K*_*α*_ = 1.739 (Si) KeV. The amount of Si is due to the substrate of pure silicon where the sample was deposited, and that of C is due to the small carbon strip with which silicon was suggested; the other elements are sodium, titanium, and oxygen which are the corresponding elements of our sample. Other traces of very small amounts of elements are left in the background noise.

### 3.4. Antibacterial Activity and Modulation of Antibiotic Activity by Na-TiNT Nanocrystals

The MICs of Na-TiNT for all strains (values ≥1024 *μ*g/mL) were considered clinically irrelevant according to the classification of Holetz and colleagues [36]. This result does not coadunate with others in the literature since titanate and titanium oxide antimicrobial activities are multiform and depend on several factors (e.g., physicochemical properties). Thus, samples of silver titanate nanotubes presented a remarkable antibiotic property against *E. coli* (ATCC 25922) and *S. aureus* (ATCC 6538); better results were observed with 100 nm-diameter nanotubes (nanotube agglomeration particles) both in the dark and under UV light. In the aforementioned study, titanium oxide nanotubes already showed antibiotic activity, although lower than those doped with silver [12].

Similarly, commercial TiO_2_ nanoparticles exhibited low bactericidal power against *E. coli* (ATCC 700926), while the transformation of this into strontium titanate ferrite metal oxide (SrTi_1−x_Fe_x_O_3−*δ*_) dramatically increased this ability [13]. Cheng and his coworkers were able to produce, from Ag-TiO_2_ nanoparticles, a composite with PVC (polyvinyl chloride) that suggests good antimicrobial activity against the *E. coli* (8099) and *S. aureus* (ATCC 6538) strains [11]. In another work, cellulose-based composite papers were produced using Ag-TiO_2_ and acid-corroded Ag-TiO_2_ nanobelt in different fractions and exhibited a good antibacterial inhibition. This is closely related to the release of silver ion from the paper [37]. Silver is not the only dopant that induces an antibiotic effect, and surfaces coated with TiO_2_(Fe^3+^) nanostructured thin films eliminated deposits of *E. coli* (ATCC 25922) after two hours under UV radiation [14]. Conversely, gold had a detrimental effect on the TiO_2_ nanosystem properties against *B. subtilis* [15]. When it comes to analyzing the antibacterial action mechanism of this type of nanoparticle, the inhibition can be supposedly due to the size of nanoparticles themselves or by releasing of metal ions. Copper and silver cations may, for example, interact with negatively charged parts of the cell wall and rupture it, leading to cell death. Nanoparticles can also destabilize the outer membrane in Gram-negative bacteria and provide membrane break, thereby causing intracellular ATP reduction [3].

Titanate nanotubes are a safe system for intestinal formulations as they are practically not absorbed from the intestine [37]. However, it is also known that ion exchange in one-dimensional titanate nanotubes (TiONTs) greatly influences their toxicity. In some cases, the incorporation of metallic ions, such as Ag and Cu ion-exchange TiONT, significantly increases the toxicity of TiONTs [38]. Nevertheless, Na-TiNT has a basic character, and the interaction between sodium titanate nanotubes and organic acids depends on the nature of the acid [35]. Na-TiNT interacts with aniline, and no chemical, morphological, or compositional change is observed [35]. A highly effective method treatment for bacterial infection in a short time is the insertion of Na^+^ in Prussian blue (PB) under microwave (MW) irradiation accelerating the release of Fe^2+^ and Fe^3+^ from PB to easily pass through the bacterial membrane to promote the Fenton reaction and glutathione (GSH) consumption in bacteria, which leads to the final death of bacteria [13].

Rónavári and colleagues screened, by the agar diffusion method, the modification of the antimicrobial capacity of ion-exchange titanate nanotubes with Ag, Mg Bi, Sb, Ca, K, Sr, Fe, and Cu, against Gram-negative (*E. coli* and *P. aeruginosa*) and Gram-positive (*M. luteus* and *B. subtilis*) microorganisms. They showed that most of the nanotubes did not obtain satisfactory results, except for Cu that inhibited *M. luteus* growth and Ag that had remarkable effects with all strains [39]. Regarding the antimicrobial effect of nanoparticles treated with the sodium element, ZnO nanostructures were found to have antimicrobial properties when doped with sodium against *E. coli* and when irradiated with UV rays [40].

Perhaps, the clinical irrelevance of our results is due to the nonuse of UV radiation in the experiments since in the work of Bessekhouad and collaborators [41], they verified the improvement of photocatalytic effects of TiO_2_ alkali metal-doped nanoparticles, among them sodium. However, discovering new substances that have antimicrobial action is important to find compounds that can help fight resistant bacteria and thus improve the action of antibiotics by reducing the amount needed to counteract infections. This effect is called “modulatory.” [42] Bacteria can be intrinsically resistant or acquire resistance by many mechanisms, including prevention of access to drug targets, changes in the structure and protection of antibiotic targets, and the direct modification or inactivation of antibiotics [43].

**Figure 5:**
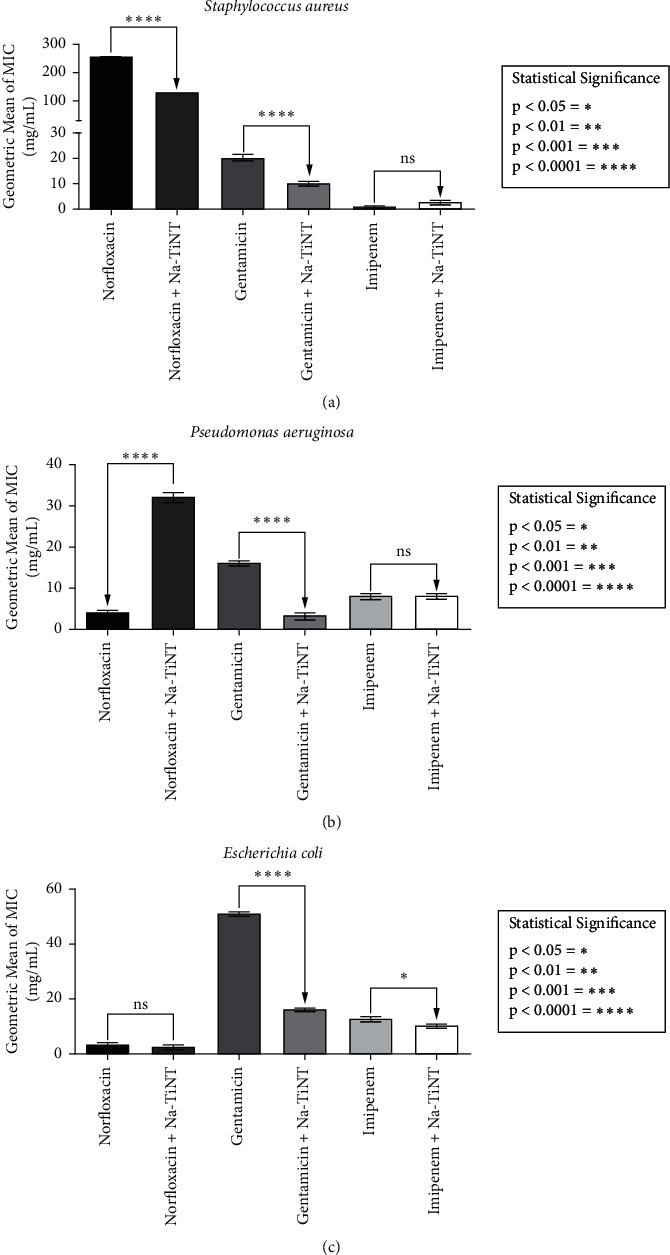
Antibiotic action of sodium titanate (Na-TiNT) in combination with different antibiotics, norfloxacin, gentamicin, and imipenem, compared to the effect of these antibiotics isolated on *Staphylococcus aureus* (a), *Pseudomonas aeruginosa* (b), and *Escherichia coli* (c). The numbers of (∗) express the level of significance between treatment and controls.

Na-TiNT nanostructures were able to access modulatory effects when used in coadministration with some antibiotics against multiresistant strains. The results are shown in Figure 5. Against Gram-positive *S. aureus* 10, the compound achieved a two-fold MIC reduction for norfloxacin and gentamicin, while no significant effect was observed with imipenem. The same synergistic behavior was reported to *β*-Ag_2_MoO_4_ microcrystals for the same antibiotics against *S. aureus* [44]. Meanwhile, for Gram-negative *P. aeruginosa* 15, only gentamicin had a synergistic effect with Na-TiNT; there was a reduction in MIC from 16 mg/mL to 3.17 mg/mL. Antagonism was identified for norfloxacin, and for imipenem, no significant variation of MIC was identified. For *E. coli* 06, a pronounced synergism was observed for gentamicin (three-fold MIC reduction), and for imipenem, the MIC decreased from 12.7 mg/mL to 10.08 mg/mL. In this case, the MIC reduction of norfloxacin was not significant.

With reference to the antibiotic gentamicin, we can see that its MIC was greatly reduced by the modulatory effect of our nanoparticle against both Gram-positive and Gram-negative. Gentamicin's traditional mode of action is recognized by inhibiting bacterial protein biosynthesis at the 30S ribosomal level or by disturbing the cell surface [45, 46]. There are several other mechanisms of antimicrobial resistance due to aminoglycosides, which include gentamicin, among them, which decreased intracellular drug concentration, target modification, enzymatic drug modification, and origin and prevalence [47]. Thus, this result suggests that Na-TiNT nanoparticles could, in principle, be an adjuvant of gentamicin against Gram-positive and Gram-negative. A weak synergism of imipenem against *E. coli* 06 has been observed. The way Gram-negative develop resistance to this type of drug is mainly by enzymatic hydrolysis [44, 45].

## 4. Conclusions

To obtain a good material with bactericidal properties, sodium titanate, Na-TiNTs, was prepared by the hydrothermal method. From the SEM and TEM images, it was concluded that the final product was a powder material formed by nanoparticles with sizes ranging from 90 to 130 nm created by an entanglement of nanotubes whose internal diameters were constant, 4 nm, but with variable outer diameters, 12 to 17 nm, showing that nanotubes can have several layers. These nanoparticles formed by nanotubes proved to be very porous, having a large surface area, which provides them with catalytic properties that contribute to antibacterial properties. Through XRD, it was found that the morphology and crystalline structure of the Na-TiNT are associated with Na_2_Ti_3_O_7_. Raman spectroscopy showed how the Na-O-Ti bonds are formed on the surfaces of nanotubes and how the octahedron of TiO_6_ is conserved even doing the doping with Na.

## Data Availability

The data used to support the findings of this study are available from the corresponding author upon request.

## References

[1] Mayers D. L., Sobel J. D., Ouellette M., Kaye K. S., Marchaim D. (2017). *Antimicrobial Drug Resistance: Clinical and Epidemiological Aspects*.

[2] Bérdy J. (2005). Bioactive microbial metabolites. *The Journal of Antibiotics*.

[3] Ruparelia J. P., Chatterjee A. K., Duttagupta S. P., Mukherji S., Mukherji S. (2008). Strain specificity in antimicrobial activity of silver and copper nanoparticles. *Acta Biomaterialia*.

[4] Kim J. S. K., Kuk E., Yu K. N. (2007). Antimicrobial effects of silver nanoparticles. *Nanomedicine: Nanotechnology, Biology and Medicine*.

[5] Sondi I., Salopek-Sondi B., Branka (2004). Silver nanoparticles as antimicrobial agent: a case study on E. Coli as A model for gram-negative bacteria. *Journal of Colloid and Interface Science*.

[6] Moura J. V. B., Freitas T. S., Cruz R. P. (2019). Antibacterial properties and modulation analysis of antibiotic activity of nace(moo4)2 microcrystals. *Microbial Pathogenesis*.

[7] Mohseni S., Aghayan M., Ghorani-Azam A., Behdani M., Asoodeh A. (2014). Evaluation of antibacterial properties of barium zirconate titanate (Bzt) nanoparticle. *Brazilian Journal of Microbiology*.

[8] Wang Z., Lee Y.-H., Wu B. (2010). Anti-microbial activities of aerosolized transition metal oxide nanoparticles. *Chemosphere*.

[9] Rekha K., Nirmala M., Nair M. G., Anukaliani A. (2010). Structural, optical, photocatalytic and antibacterial activity of zinc oxide and manganese doped zinc oxide nanoparticles. *Physica B: Condensed Matter*.

[10] Zollfrank C., Gutbrod K., Wechsler P., Guggenbichler J. P. (2012). Antimicrobial activity of transition metal acid Moo3 prevents microbial growth on material surfaces. *Materials Science and Engineering: C*.

[11] Cheng Q., Li C., Pavlinek V., Saha P., Wang H. (2006). Surface-modified antibacterial Tio2/Ag+ nanoparticles: preparation and properties. *Applied Surface Science*.

[12] Zhao C., Feng B., Li Y., Tan J., Lu X., Weng J. (2013). Preparation and antibacterial activity of titanium nanotubes loaded with Ag nanoparticles in the dark and under the Uv light. *Applied Surface Science*.

[13] Zhang L. T., Tan P. Y., Chow C. L. (2014). Antibacterial activities of mechanochemically synthesized perovskite strontium titanate ferrite metal oxide. *Colloids and Surfaces A: Physicochemical and Engineering Aspects*.

[14] Trapalis C. C., Keivanidis P., Kordas G. (2003). Tio2(Fe3+) nanostructured thin films with antibacterial properties. *Thin Solid Films*.

[15] Armelao L., Barreca D., Bottaro G. (2007). Photocatalytic and antibacterial activity of Tio2and Au/Tio2nanosystems. *Nanotechnology*.

[16] Zhao Z., Fan J., Wang J., Li R. (2012). Effect of heating temperature on photocatalytic reduction of CO2 by N-TiO2 nanotube catalyst. *Catalysis Communications*.

[17] Asapu R., Palla V. M., Wang B., Guo Z., Sadu R., Chen D. H. (2011). Phosphorus-doped titania nanotubes with enhanced photocatalytic activity. *Journal of Photochemistry and Photobiology A: Chemistry*.

[18] Liu H., Liu G., Zhou Q. (2011). Preparation and photocatalytic activity of Gd3+-doped trititanate nanotubes. *Microporous and Mesoporous Materials*.

[19] Gong D., Ho W. C. J., Tang Y. (2012). Silver decorated titanate/titania nanostructures for efficient solar driven photocatalysis. *Journal of Solid State Chemistry*.

[20] Kasuga T., Hiramatsu M., Hoson A., Sekino T., Niihara K. (1998). formation of titanium oxide nanotube. *Langmuir*.

[21] Wang L., Liu W., Wang T., Ni J. (2013). Highly efficient adsorption of Cr(vi) from aqueous solutions by amino-functionalized titanate nanotubes. *Chemical Engineering Journal*.

[22] Viana B. C., Ferreira O. P., Filho A. G. S. (2011). Alkali metal intercalated titanate nanotubes: a vibrational spectroscopy study. *Vibrational Spectroscopy*.

[23] Marques T. M. F., Sales D. A., Silva L. S. (2020). Amino-functionalized titanate nanotubes for highly efficient removal of anionic dye from aqueous solution. *Applied Surface Science*.

[24] Clsi (2015). *Methods for Dilution Antimicrobial Susceptibility Tests for Bacteria that Grow Aerobically; Approved Standard — Clsi Document M07-A10*.

[25] Javadpour M. M., Juban M. M., Lo W.-C. J. (1996). De novo antimicrobial peptides with low mammalian cell toxicity. *Journal of Medicinal Chemistry*.

[26] Coutinho H. D. M., Costa J. G. M., Siqueira-Júnior J. P., Lima E. O. (2008). In vitro anti-staphylococcal activity of Hyptis martiusii Benth against methicillin-resistant *Staphylococcus aureus*: MRSA strains. *Revista Brasileira de Farmacognosia*.

[27] Li X., Li W., Li M. (2015). Glucose-assisted synthesis of the hierarchical Tio2 Nanowire@Mos2 nanosheet nanocomposite and its synergistic lithium storage performance. *Journal of Materials Chemistry A*.

[28] Gusmão, Suziete B. S., Ghosh A. (2019). One-pot synthesis of titanate nanotubes decorated with anatase nanoparticles using a microwave-assisted hydrothermal reaction. *Journal of Nanomaterials*.

[29] Ferreira O. P., Souza Filho A. G., Mendes Filho J., Alves O. L. (2006). Unveiling the structure and composition of titanium oxide nanotubes through ion exchange chemical reactions and thermal decomposition processes. *Journal of the Brazilian Chemical Society*.

[30] Marques T. M. F., Luz-Lima C., Sacilloti M. (2017). Photoluminescence enhancement of titanate nanotubes by insertion of rare earth ions in their interlayer spaces. *Journal of Nanomaterials*.

[31] Lee S. S., Byeon S. H. (2004). Structural and morphological behavior of TiO_2_ rutile obtained by hydrolysis reaction of Na2ti3o7. *Bulletin of the Korean Chemical Society*.

[32] Freitas T. S., Marques T. M. F., Barros L. N. L. C. (2021). Synthesis of Cu-tint, characterization, and antibacterial properties evaluation. *Materials Today Chemistry*.

[33] Zheng Z., Jia J., Zhong Z. (2014). Revisiting the Co oxidation reaction on various Au/Tio2 catalysts: roles of the surface oh groups and the reaction mechanism. *Journal of Nanoscience and Nanotechnology*.

[34] Bavykin D. V., Walsh F. C. (2010). *Titanate and Titania Nanotubes: Synthesis, Properties and Applications*.

[35] Rodrigues C. M., Ferreira O. P., Alves O. L. (2010). Interaction of sodium titanate nanotubes with organic acids and Base: chemical, structural and morphological stabilities. *Journal of the Brazilian Chemical Society*.

[36] Holetz F. B., Pessini G. L., Sanches N. R., Cortez D. A. G., Nakamura C. V., Dias Filho B. P. (2002). Screening of some plants used in the Brazilian folk medicine for the treatment of infectious diseases. *Memórias do Instituto Oswaldo Cruz*.

[37] Wang J., Liu W., Li H. (2013). Preparation of cellulose fiber-TiO2 nanobelt-silver nanoparticle hierarchically structured hybrid paper and its photocatalytic and antibacterial properties. *Chemical Engineering Journal*.

[38] Fenyvesi F., Kónya Z., Rázga Z. (2014). Investigation of the cytotoxic effects of titanate nanotubes on caco-2 cells. *AAPS PharmSciTech*.

[39] Rónavári A., Kovács D., Vágvölgyi C., Kónya Z., Kiricsi M., Pfeiffer I. (2016). Ion exchange defines the biological activity of titanate nanotubes. *Journal of Basic Microbiology*.

[40] Wu C., Shen L., Huang Q., Zhang Y.-C. (2011). Synthesis of Na-doped zno nanowires and their antibacterial properties. *Powder Technology*.

[41] Bessekhouad Y., Robert D., Weber J.-V., Chaoui N. (2004). Effect of alkaline-doped Tio2 on photocatalytic efficiency. *Journal of Photochemistry and Photobiology A: Chemistry*.

[42] Tintino S. R., Morais-Tintino C. D., Campina F. F. (2016). Action of cholecalciferol and alpha-tocopherol on Staphylococcus aureus efflux pumps. *EXCLI Journal*.

[43] Blair J. M. A., Webber M. A., Baylay A. J., Ogbolu D. O., Piddock L. J. V. (2015). Molecular mechanisms of antibiotic resistance. *Nature Reviews Microbiology*.

[44] Moura J. V. B., Freitas T. S., Cruz R. P. (2017). *β*-Ag2MoO4 microcrystals: characterization, antibacterial properties and modulation analysis of antibiotic activity. *Biomedicine & Pharmacotherapy*.

[45] Kadurugamuwa J. L., Clarke A. J., Beveridge T. J. (1993). Surface action of gentamicin on Pseudomonas aeruginosa. *Journal of Bacteriology*.

[46] Hahn F. E., Sarre S. G. (1969). Mechanism of action of gentamicin. *The Journal of infectious diseases*.

[47] Magnet S., Blanchard J. S. (2005). Molecular insights into aminoglycoside action and resistance. *Chemical Reviews*.

